# Oxidation and reduction data of subphthalocyanines

**DOI:** 10.1016/j.dib.2019.105039

**Published:** 2019-12-24

**Authors:** Pieter J. Swarts, Jeanet Conradie

**Affiliations:** Department of Chemistry, PO Box 339, University of the Free State, Bloemfontein, 9300, South Africa

**Keywords:** Subphthalocyanines, Cyclic voltammetry, Oxidation, Reduction

## Abstract

The data presented in this paper are related to the research article entitled “*Electrochemical behaviour of chloro- and hydroxy-subphthalocyanines*” [1]. This paper presents detailed oxidation and reduction potential data, obtained from cyclic voltammograms of three subphthalocyanines (SubPcs), in both dichloromethane (DCM) and dichloroethane (DCE) as solvent. The first SubPc is the unsubstituted boron-subphthalocyanine, (ClB)SubPc(H)_12_, as reference SubPc, the second SubPc is (ClB)SubPc(F)_12_, containing an electron-poor macro-cycle and (HOB)SubPc(C_12_H_25_)_6_(H)_6_, containing an electron-rich macro-cycle. The oxidation and reduction potential of (ClB)SubPc(F)_12_ in DCM is ca. 0.5 V more positive than that of the reference ClBSubPc(H)_12_, while oxidation and reduction potential of (HOB)SubPc(C_12_H_25_)_6_(H)_6_ in DCM is ca. 0.45 V more negative than that of the reference (ClB)SubPc(H)_12_.

**Table:** Specifications Table

Subject	Chemistry
Specific subject area	Electrochemistry
Type of data	Table Image Graph Figure
How data were acquired	Princeton Applied Research PARSTAT 2273 potentiostat running Powersuite software (Version 2.58).
Data format	Raw Analyzed
Parameters for data collection	Samples was used as synthesized. All the electrochemical experiments were performed in an M Bruan Lab Master SP glove box under a high purity argon atmosphere (H_2_O and O_2_ < 10 ppm).
Description of data collection	All electrochemical experiments were done in a 2 ml electrochemical cell containing three-electrodes (a glassy carbon working electrode, a Pt auxiliary electrode and a Pt pseudo reference electrode), connected to a Princeton Applied Research PARSTAT 2273 electrochemical analyzer. Data obtained were exported to excel for analysis and diagram preparation.
Data source location	Institution: University of the Free State City/Town/Region: Bloemfontein Country: South Africa
Data accessibility	With the article
Related research article	P.J. Swarts, J. Conradie, Electrochemical behaviour of chloro- and hydroxy- subphthalocyanines, Electrochimica Acta https://doi.org/10.1016/j.electacta.2019.135165

**Table:** 

**Value of the Data** • This data provides cyclic voltammograms and detailed electrochemical data for three subphthalocyanines for scan rates over two orders of magnitude (0.05–5.0 Vs^−1^). • This data illustrates the influence of the solvent on the resolution of the cyclic voltammograms for three subphthalocyanines. • This data illustrates the influence of the solvent on the value of the redox potentials for three subphthalocyanines. • This data illustrates the influence of electron donating and electron withdrawing substituents on the redox potential of the subphthalocyanine. • This data illustrates that electrochemical quasi reversible oxidation can be obtained when electrochemical experiments are performed in a high purity argon atmosphere, while using DCM or DCE as the solvent and [N(^*n*^Bu)_4_][B(C_6_F_5_)_4_] as supporting electrolyte.

## 1 Data description

**Fig. 1:**
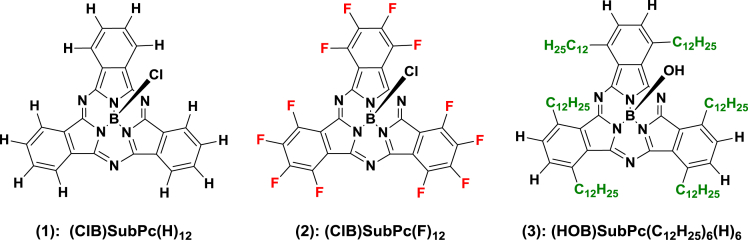
Structure of the SubPcs 1–3.

**Fig. 2:**
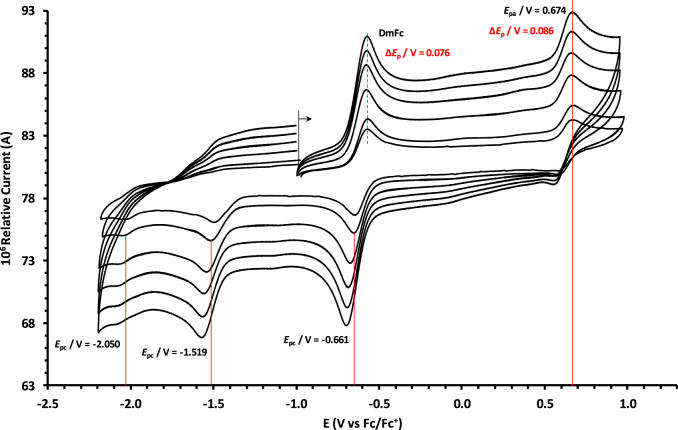
Cyclic voltammograms in DCM of (ClB)SubPc(H)_12_, 1, at scan rates 0.050 (smallest peak currents), 0.100, 0.200, 0.300, 0.400 and 0.500 (largest peak currents). All scans initiated in the positive direction. Wave I is the oxidation and waves II and III are reduction of (ClB)SubPc(H)_12_. Data of 0.100 V s^−1^ shown on graph.

**Fig. 3:**
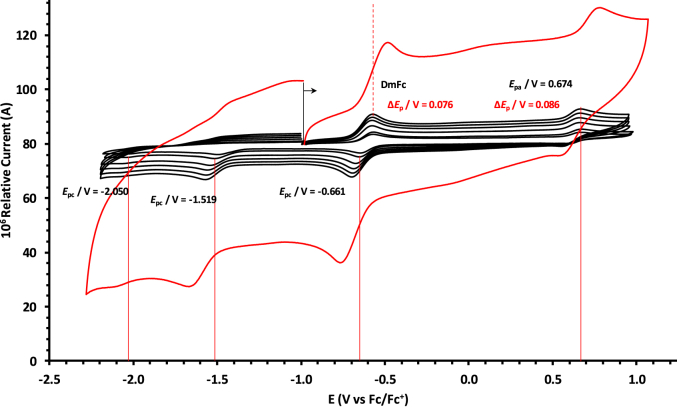
Cyclic voltammograms in DCM of (ClB)SubPc(H)_12_, 1, scan rates 0.050 (smallest peak currents), 0.100, 0.200, 0.300, 0.400, 0.500 and 5.000 Vs^−1^ (largest peak currents shown in red). All scans initiated in the positive direction. Wave I is the oxidation and waves II and III are reduction of (ClB)SubPc(H)_12_. Data of 0.100 V s^−1^ shown on graph.

**Fig. 4:**
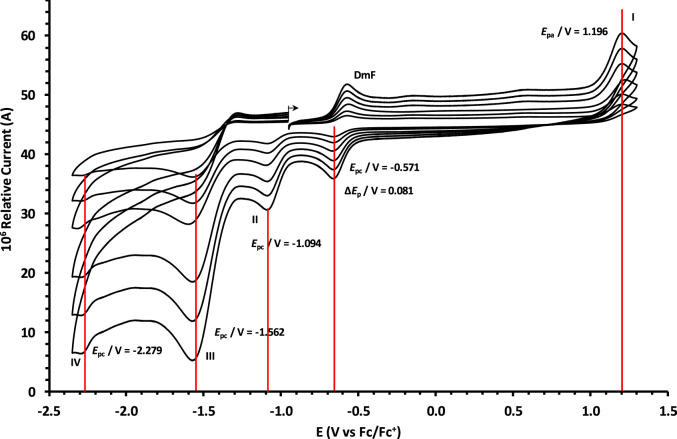
Cyclic voltammograms in DCM of (ClB)SubPc(F)_12_, 2, at scan rates 0.050 (smallest peak currents), 0.100, 0.200, 0.300, 0.400 and 0.500 (largest peak currents). All scans initiated in the positive direction. Wave I is the oxidation and waves II, III and IV are reduction of (ClB)SubPc(F)_12_. Data of 0.100 V s^−1^ shown on graph.

**Fig. 5:**
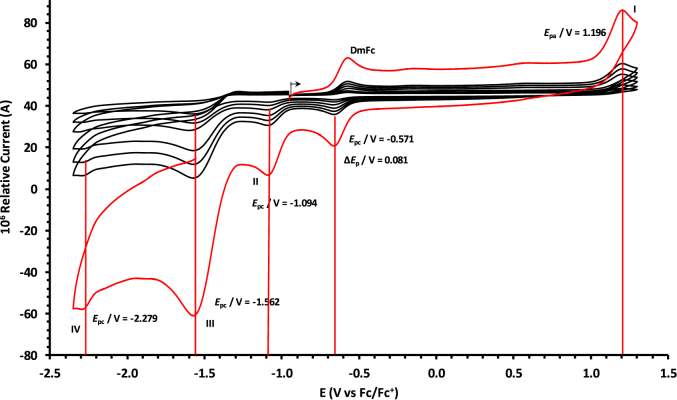
Cyclic voltammograms in DCM of (ClB)SubPc(F)_12_, 2, at scan rates 0.050 (smallest peak currents), 0.100, 0.200, 0.300, 0.400, 0.500 and 5.000 Vs^−1^ (largest peak currents shown in red). All scans initiated in the positive direction. Wave I is the oxidation and waves II, III and IV are reduction of (ClB)SubPc(F)_12_. Data of 0.100 V s^−1^ shown on graph.

**Fig. 6:**
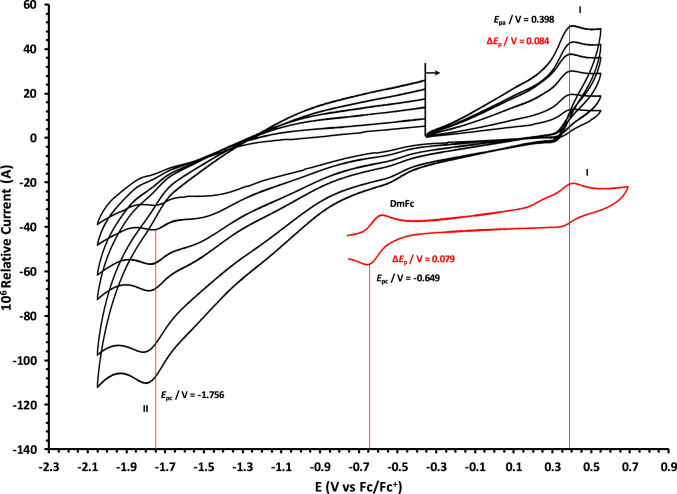
Cyclic voltammograms in DCM of (HOB)SubPc(C_12_H_25_)_6_(H)_6_, 3, at scan rates 0.050 (smallest peak currents), 0.100, 0.200, 0.300, 0.400 and 0.500 (largest peak currents). All scans initiated in the positive direction. Wave I is the oxidation and wave II is the reduction of (HOB)SubPc(C_12_H_25_)_6_(H)_6_. Data of 0.100 V s^−1^ shown on graph.

**Fig. 7:**
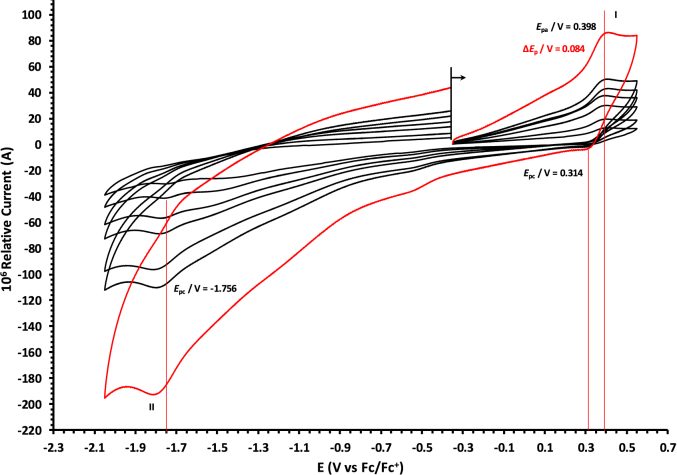
Cyclic voltammograms in DCM of (HOB)SubPc(C_12_H_25_)_6_(H)_6_, 3, at scan rates 0.050 (smallest peak currents), 0.100, 0.200, 0.300, 0.400, 0.500 and 5.000 Vs^−1^ (largest peak currents shown in red). All scans initiated in the positive direction. Wave I is the oxidation and wave II is the reduction of (HOB)SubPc(C_12_H_25_)_6_(H)_6_. Data of 0.100 V s^−1^ shown on graph.

**Fig. 8:**
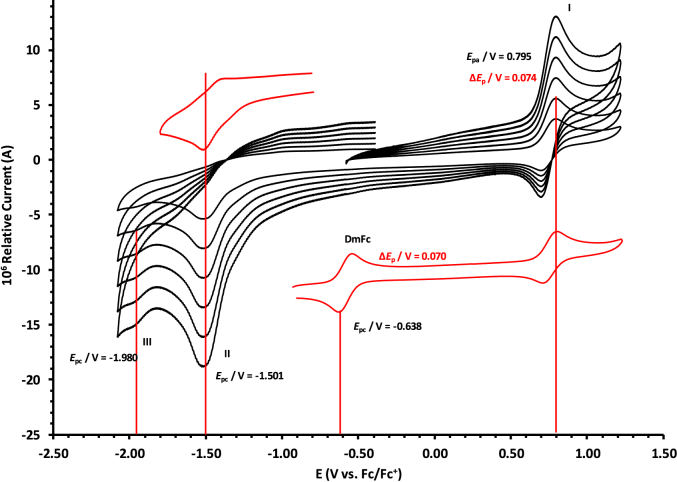
Cyclic voltammograms in DCE of (ClB)SubPc(H)_12_, 1, at scan rates 0.050 (smallest peak currents), 0.100, 0.200, 0.300, 0.400 and 0.500 (largest peak currents). All scans initiated in the positive direction. Wave I is the oxidation and waves II and III are reduction of (ClB)SubPc(H)_12_. Data of 0.100 V s^−1^ shown on graph.

**Fig. 9:**
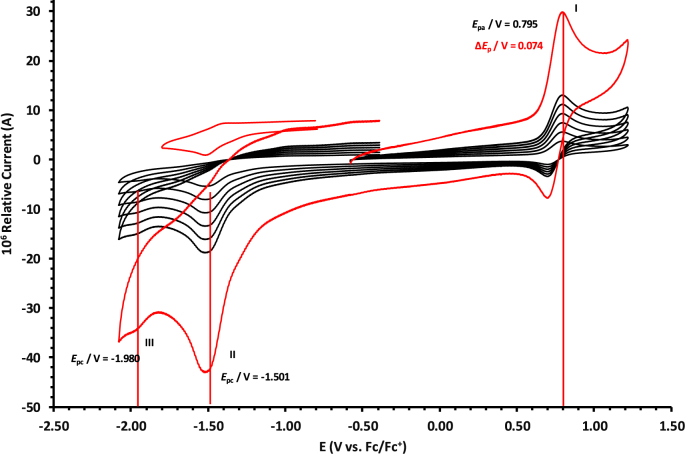
Cyclic voltammograms in DCE of (ClB)SubPc(H)_12_, 1, scan rates 0.050 (smallest peak currents), 0.100, 0.200, 0.300, 0.400, 0.500 and 5.000 Vs^−1^ (largest peak currents shown in red). All scans initiated in the positive direction. Wave I is the oxidation and waves II and III are reduction of (ClB)SubPc(H)_12_. Data of 0.100 V s^−1^ shown on graph.

**Fig. 10:**
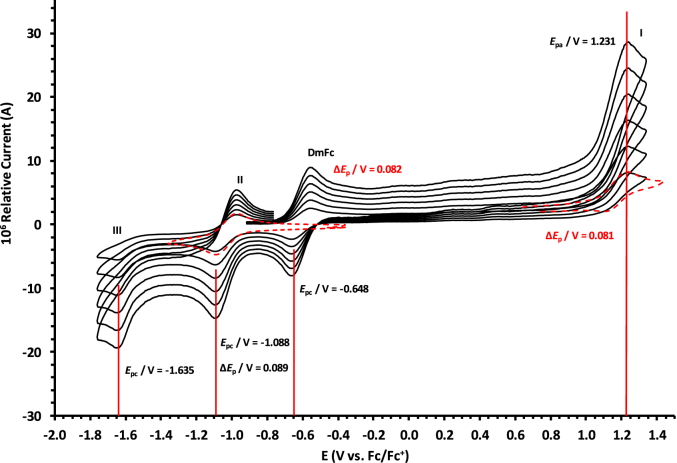
Cyclic voltammograms in DCE of (ClB)SubPc(F)_12_, 2, at scan rates 0.050 (smallest peak currents), 0.100, 0.200, 0.300, 0.400 and 0.500 (largest peak currents). All scans initiated in the positive direction. Wave I is the oxidation and waves II and III are reduction of (ClB)SubPc(F)_12_. Data of 0.100 V s^−1^ shown on graph. Dotted lines are 0.050 V s^−1^.

**Fig. 11:**
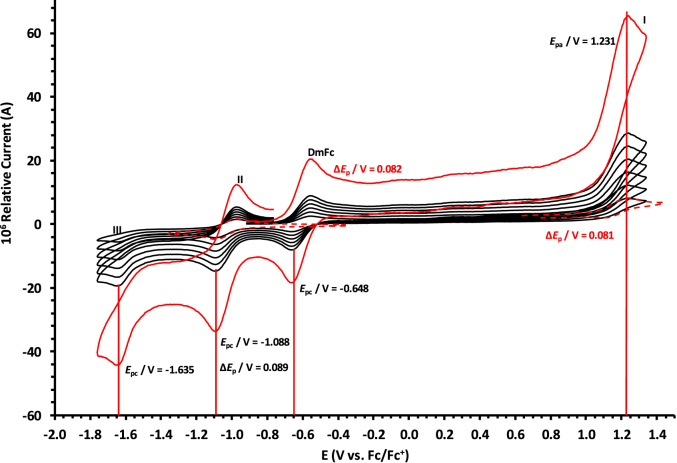
Cyclic voltammograms in DCE of (ClB)SubPc(F)_12_, 2, at scan rates 0.050 (smallest peak currents), 0.100, 0.200, 0.300, 0.400, 0.500 and 5.000 Vs^−1^ (largest peak currents shown in red). All scans initiated in the positive direction. Wave I is the oxidation and waves II and III are reduction of (ClB)SubPc(F)_12_. Data of 0.100 V s^−1^ shown on graph. Dotted lines are 0.050 V s^−1^.

**Fig. 12:**
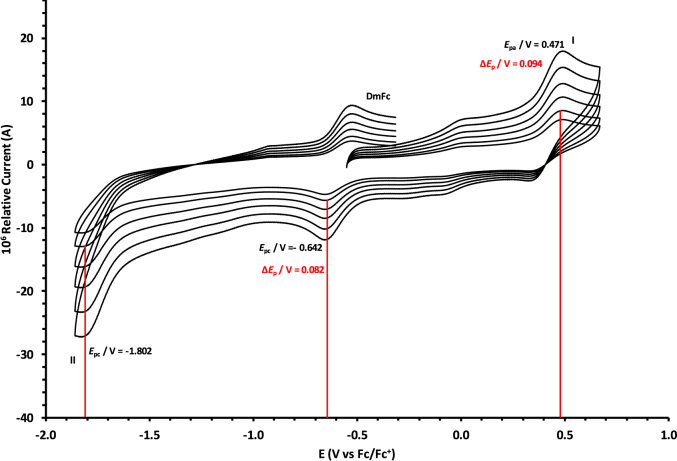
Cyclic voltammograms in DCE of (HOB)SubPc(C_12_H_25_)_6_(H)_6_, 3, at scan rates 0.050 (smallest peak currents), 0.100, 0.200, 0.300, 0.400 and 0.500 (largest peak currents). All scans initiated in the positive direction. Wave I is the oxidation and wave II is the reduction of (HOB)SubPc(C_12_H_25_)_6_(H)_6_. Data of 0.100 V s^−1^ shown on graph.

**Fig. 13:**
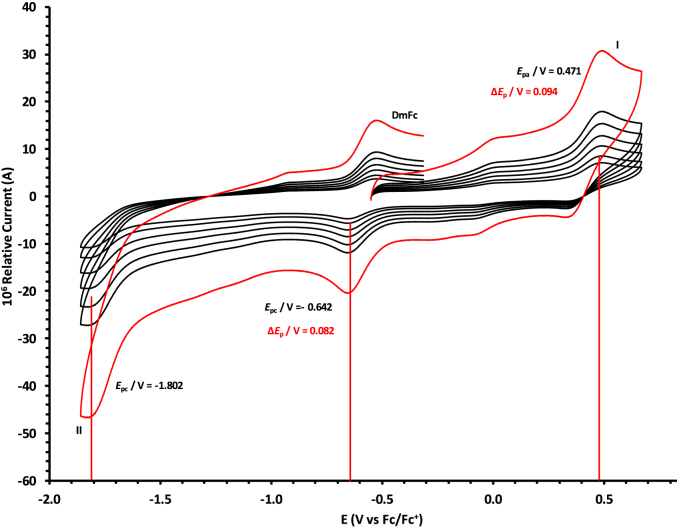
Cyclic voltammograms in DCE of (HOB)SubPc(C_12_H_25_)_6_(H)_6_, 3, at scan rates 0.050 (smallest peak currents), 0.100, 0.200, 0.300, 0.400, 0.500 and 5.000 Vs^−1^ (largest peak currents shown in red). All scans initiated in the positive direction. Wave I is the oxidation and wave II is the reduction of (HOB)SubPc(C_12_H_25_)_6_(H)_6_. Data of 0.100 V s^−1^ shown on graph.

The oxidation and reduction potential data of the unsubstituted boron-subphthalocyanine, (ClB)SubPc(H)_12_, **1**, as reference SubPc, (ClB)SubPc(F)_12_, **2**, containing an electron-poor macro-cycle and (HOB)SubPc(C_12_H_25_)_6_(H)_6_, **3**, containing an electron-rich macro-cycle, is presented here. Fig. 1 shows the structures of the SubPcs **1**–**3**. Cyclic voltammograms and redox data obtained in dichloromethane (DCM) as solvent are given in Fig. 2, Fig. 3, Fig. 4, Fig. 5, Fig. 6, Fig. 7 and Table 1, Table 2, Table 3 respectively. Cyclic voltammograms and redox data obtained in dichloroethane (DCE) as solvent are given in Fig. 8, Fig. 9, Fig. 10, Fig. 11, Fig. 12, Fig. 13 and Table 4, Table 5, Table 6 respectively. The 0.10 Vs^−1^ scans and data are from the research article related to this article “*Electrochemical behaviour of chloro- and hydroxy-subphthalocyanines*” [1]. The CV scan indicated in red in selected graphs are done at 5.00 V s^−1^. The oxidation and reduction potential data obtained here, compare well with available published data on obtained under different experimental conditions (namely different solvents, scan rates and supporting electrolytes) for SubPc **1** [[2], [3], [4], [5], [6], [7]] and SubPc **2** [8]. No detail electrochemical data is available for SubPc **3**. Data presented in this study for **1** and **3** in DCM, and **1**–**3** in DCE show electrochemical quasi reversible oxidation. No electrochemical quasi reversible oxidation with peak current ratios = 1 and peak current separation <0.09 V, is reported till date for SubPcs [3,4].

**Table 1:** Electrochemical data (potential in V *vs* Fc/Fc^+^) in DCM for *c.a*. 0.0005 mol dm^−3^ of (ClB)SubPc(H)_12_, 1, at indicated scan rates (*v* in V/s).

*v* (V/s)	*E*_pa_/V	Δ*E*_p_/V	*E*^o^′/V	*i*_pa_/μA	*i*_pc_/*i*_pa_
**Wave: I**					
0.050	0.673	0.084	0.627	2.02	0.99
**0.100**	**0.674**	**0.086**	**0.628**	**3.08**	**0.99**
0.200	0.679	0.089	0.628	4.84	0.99
0.300	0.680	0.092	0.628	6.13	0.99
0.400	0.687	0.095	0.629	7.49	0.99
0.500	0.692	0.099	0.629	8.25	0.99
5.000	0.707	–	–	–	–
**Wave: II**					
0.050	−1.516	–	–	2.11	–
**0.100**	**−1.519**	-	-	**3.08**	-
0.200	−1.532	–	–	4.35	–
0.300	−1.544	–	–	6.15	–
0.400	−1.566	–	–	7.49	–
0.500	−1.574	–	–	8.34	–
5.000	−1.592	–	–	–	–
**Wave: III**					
0.050	−2.050	–	–	–	–
**0.100**	**−2.050**	-	-	-	-
0.200	−2.051	–	–	–	–
0.300	−2.051	–	–	–	–
0.400	−2.051	–	–	–	–
0.500	−2.051	–	–	–	–
5.000	−2.055	–	–	–	–

Data for 0.100 V/s shown in bold font.

**Table 2:** Electrochemical data (potential in V *vs* Fc/Fc^+^) in DCM for *c.a*. 0.0005 mol dm^−3^ of (ClB)SubPc(F)_12_, 2, at indicated scan rates.

*v* (V/s)	*E*_pa_/V	Δ*E*_p_/V	*E*^o^′/V	*i*_pa_/μA	*i*_pc_/*i*_pa_
**Wave: I**					
0.050	1.196	–	–	1.99	–
**0.100**	**1.196**	-	-	**3.21**	-
0.200	1.196	–	–	4.98	–
0.300	1.197	–	–	6.21	–
0.400	1.197	–	–	8.11	–
0.500	1.197	–	–	9.00	–
5.000	1.207	–	–	–	–
**Wave: II**					
0.050	−1.093	–	–	1.95	–
**0.100**	**−1.094**	**0.088**	**−1.050**	**3.22**	**0.97**
0.200	−1.098	–	–	4.58	–
0.300	−1.101	–	–	6.15	–
0.400	−1.108	–	–	8.78	–
0.500	−1.110	–	–	9.25	–
5.000	−1.119	–	–	–	–
**Wave: III**					
0.050	−1.560	–	–	–	–
**0.100**	**−1.562**	-	-	-	-
0.200	−1.564	–	–	–	–
0.300	−1.567	–	–	–	–
0.400	−1.568	–	–	–	–
0.500	−1.570	–	–	–	–
5.000	−1.581	–	–	–	–
**Wave: IV**					
0.050	−2.276	–	–	–	–
**0.100**	**−2.279**	-	-	-	-
0.200	−2.284	–	–	–	–
0.300	−2.288	–	–	–	–
0.400	−2.290	–	–	–	–
0.500	−2.292	–	–	–	–
5.000	−2.311	–	–	–	–

Data for 0.100 V/s shown in bold font.

**Table 3:** Electrochemical data (potential in V *vs* Fc/Fc^+^) in DCM for *c.a*. 0.002 mol dm^−3^ of (HOB)SubPc(C_12_H_25_)_6_(H)_6_, 3, at indicated scan rates.

*v* (V/s)	*E*_pa_/V	Δ*E*_p_/V	*E*^o^′/V	*i*_pa_/μA	*i*_pc_/*i*_pa_
**Wave: I**					
0.050	0.398	0.082	0.355	2.22	–
**0.100**	**0.398**	**0.084**	**0.356**	**3.46**	-
0.200	0.399	0.086	0.356	4.98	–
0.300	0.401	0.088	0.356	6.23	–
0.400	0.402	0.090	0.358	8.01	–
0.500	0.402	0.092	0.359	9.11	–
5.000	0.405	–	–	–	–
**Wave: II**					
0.050	−1.752	–	–	–	–
**0.100**	**−1.756**	-	-	-	-
0.200	−1.762	–	–	–	–
0.300	−1.769	–	–	–	–
0.400	−1.772	–	–	–	–
0.500	−1.780	–	–	–	–
5.000	−1.792	–	–	–	–

Data for 0.100 V/s shown in bold font.

**Table 4:** Electrochemical data (potential in V *vs* Fc/Fc^+^) in DCE for *c.a*. 0.0005 mol dm^−3^ of (ClB)SubPc(H)_12_, 1, at indicated scan rates (*v* in V/s).

*v* (V/s)	*E*_pa_/V	Δ*E*_p_/V	*E*^o^′/V	*i*_pa_/μA	*i*_pc_/*i*_pa_
**Wave: I**					
0.050	0.795	0.072	0.757	2.14	0.99
**0.100**	**0.795**	**0.074**	**0.758**	**3.75**	**0.99**
0.200	0.796	0.076	0.758	4.95	0.99
0.300	0.796	0.078	0.759	6.11	0.99
0.400	0.797	0.081	0.759	7.95	0.99
0.500	0.798	0.082	0.759	8.95	0.99
5.000	0.808	–	–	–	–
**Wave: II**					
0.050	−1.501	–	–	2.36	–
**0.100**	**−1.501**	-	-	**3.75**	-
0.200	−1.502	–	–	4.92	–
0.300	−1.502	–	–	6.44	–
0.400	−1.503	–	–	7.98	–
0.500	−1.504	–	–	10.62	–
5.000	−1.505	–	–	–	–
**Wave: III**					
0.050	−1.980	–	–	–	–
**0.100**	**−1.980**	-	-	-	-
0.200	−1.980	–	–	–	–
0.300	−1.980	–	–	–	–
0.400	−1.980	–	–	–	–
0.500	−1.980	–	–	–	–
5.000	−1.980	–	–	–	–

Data for 0.100 V/s shown in bold font.

**Table 5:** Electrochemical data (potential in V *vs* Fc/Fc^+^) in DCE for *c.a*. 0.0005 mol dm^−3^ of (ClB)SubPc(F)_12_, 2, at indicated scan rates.

*v* (V/s)	*E*_pa_/V	Δ*E*_p_/V	*E*^o^′/V	*i*_pa_/μA	*i*_pc_/*i*_pa_
**Wave: I**					
0.050	1.230	0.080	1.190	2.18	0.99
**0.100**	**1.231**	**0.081**	**1.190**	**3.75**	**0.99**
0.200	1.232	0.083	1.191	5.24	0.99
0.300	1.233	0.084	1.191	6.29	0.99
0.400	1.234	0.085	1.191	8.65	0.99
0.500	1.235	0.087	1.191	9.54	0.99
5.000	–	–	–	–	–
**Wave: II**					
0.050	−1.087	0.088	−1.043	2.90	0.99
**0.100**	**−1.088**	**0.089**	**−1.044**	**3.80**	**0.99**
0.200	−1.089	0.091	−1.044	5.25	0.99
0.300	−1.092	0.093	−1.045	7.32	0.99
0.400	−1.092	0.094	−1.045	8.55	0.99
0.500	−1.094	0.096	−1.046	10.60	0.99
5.000	–	–	–	–	–
**Wave: III**					
0.050	−1.634	–	–	2.89	–
**0.100**	**−1.635**	–	–	**3.65**	–
0.200	−1.636	–	–	5.43	–
0.300	−1.637	–	–	7.38	–
0.400	−1.639	–	–	8.74	–
0.500	−1.641	–	–	10.12	–
5.000	–	–	–	–	–

Data for 0.100 V/s shown in bold font.

**Table 6:** Electrochemical data (potential in V *vs* Fc/Fc^+^) in DCE for *c.a*. 0.002 mol dm^−3^ of (HOB)SubPc(C_12_H_25_)_6_(H)_6_, 3, at indicated scan rates.

*v* (V/s)	*E*_pa_/V	Δ*E*_p_/V	*E*^o^′/V	*i*_pa_/μA	*i*_pc_/*i*_pa_
**Wave: I**					
0.050	0.471	0.093	0.424	2.34	0.92
**0.100**	**0.471**	**0.094**	**0.426**	**3.98**	**0.94**
0.200	0.471	0.095	0.426	4.95	0.95
0.300	0.472	0.095	0.427	6.12	0.95
0.400	0.472	0.096	0.428	7.42	0.96
0.500	0.482	0.097	0.428	7.95	0.96
5.000	0.493	–	–	–	–
**Wave: II**					
0.050	−1.801	–	–	–	–
**0.100**	**−1.804**	–	–	–	–
0.200	−1.811	–	–	–	–
0.300	−1.815	–	–	–	–
0.400	−1.821	–	–	–	–
0.500	−1.834	–	–	–	–
5.000	−1.844	–	–	–	–

Data for 0.100 V/s shown in bold font.

## 2 Experimental design, materials, and methods

Electrochemical studies by means of cyclic voltammetry (CV) experiments were performed in an M Bruan Lab Master SP glove box under a high purity argon atmosphere (H_2_O and O_2_ < 10 ppm), utilizing a Princeton Applied Research PARSTAT 2273 potentiostat running Powersuite software (Version 2.58).

The cyclic voltammetry experimental setup consists of a cell with three electrodes, namely (i) a glassy carbon electrode as working electrode, (ii) a platinum wire auxiliary and (ii) a platinum wire as pseudo reference electrode. The glassy carbon working electrode was polished and prepared before every experiment on a Buhler polishing mat first with 1-micron and then with ¼-micron diamond paste, rinsed with H_2_O, acetone and DCM, and dried before each experiment.

Electrochemical analysis in dichloromethane (DCM, anhydrous, ≥ 99.8%, contains 40–150 ppm amylene as stabilizer) as solvent was at RT and in dichloroethane (DCE, anhydrous, 99.8%) at 60 °C. The analyte solutions in DCM as solvent were: 0.0005 M for (ClB)SubPc(H)_12_, 1, 0.0005 M for (ClB)SubPc(F)_12_, 2, and 0.004 mol dm^−3^ for (SubPc 3). The analyte solutions in DCE as solvent were: 0.0005 M for (ClB)SubPc(H)_12_, 1, 0.0005 M for (ClB)SubPc(F)_12_, 2, and 0.004 mol dm^−3^ for (SubPc 3). The supporting electrolyte 0.1 mol dm^−3^ (in DCM) or 0.2 mol dm^−3^ (in DCE) tetrabutylammonium tetrakispentafluorophenylborate [N(^*n*^Bu)_4_][B(C_6_F_5_)_4_] [9].

**Fig. 14:**
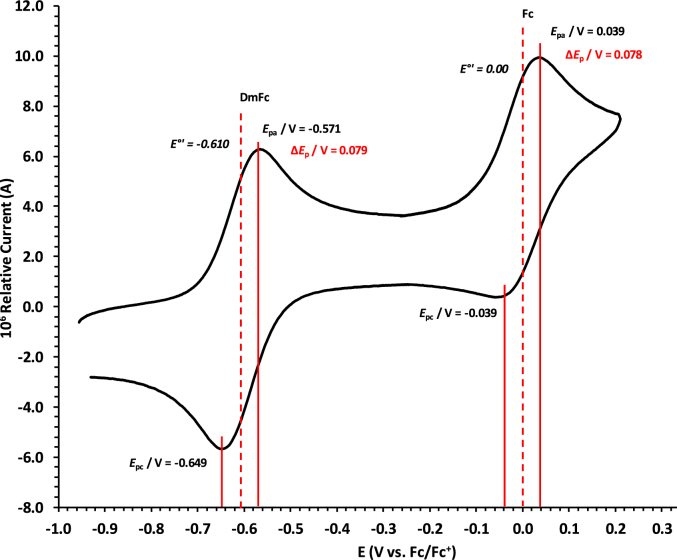
Cyclic voltammograms in DCM of Decamethylferrocene at scan rate 0.100 Vs^−1^. All scans initiated in the positive direction. Data of 0.100 V s^−1^ shown on graph.

**Fig. 15:**
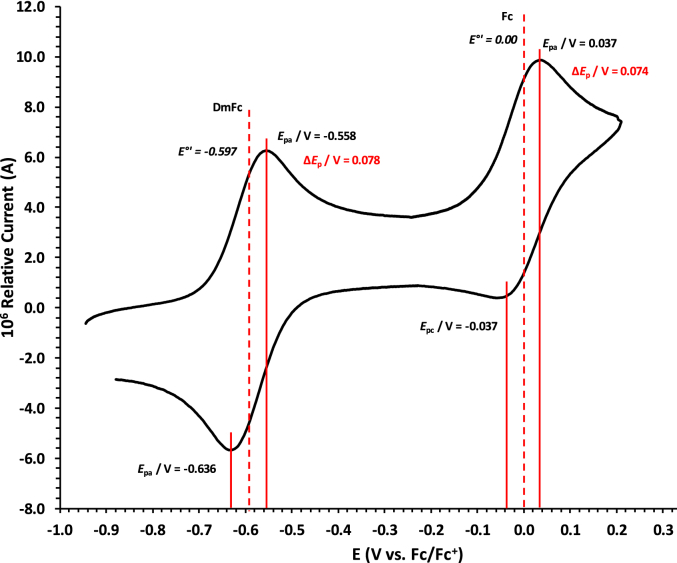
Cyclic voltammograms in DCE of Decamethylferrocene at scan rate 0.100 Vs^−1^. All scans initiated in the positive direction. Data of 0.100 V s^−1^ shown on graph.

Experimental potential data was measured vs. the redox couple of decamethyl ferrocene DmFc as internal standard and reported vs. the redox couple of ferrocene Fc, as suggested by IUPAC [10]. Under our experimental conditions E(DmFc/DmFc+ = − 0.610 V Fc/Fc^+^ (DCM) and −0.597 V Fc/Fc^+^ (DCE) (see Fig. 14, Fig. 15). Scan rates were done over two orders of magnitude, namely between 0.05 and 5.00 Vs^−1^.

## Acknowledgments

This work has received support from the South African National Research Foundation (Grant numbers 113327 and 96111) and the Central Research Fund of the University of the Free State, Bloemfontein, South Africa.

## Conflict of Interest

The authors declare that they have no known competing financial interests or personal relationships that could have appeared to influence the work reported in this paper.
